# Value of Oral Hygiene Lectures

**Published:** 1916-09

**Authors:** Thaddeus P. Hyatt

**Affiliations:** New York


### THE VALUE OF ORAL HYGIENE LECTURES TO THE PUBLIC AND THE RESPONSIBILITY OF THE DENTAL PROFESSION IN RELATION TO THIS WORK.

THADDEUS P. HYATT, D.D.S., NEW YORK.

Before dental colleges were established it was the custom among dentists to keep what knowledge they had acquired to themselves, or to impart it to their pupils only. With the establishment of dental colleges and dental societies, a broader view and a more liberal feeling came into play. Men became willing to share with other members of their profession the discoveries they had made, and as respect and honor were given to them, others strived to obtain honors by study and research work. The gaining of a deeper insight into the anatomy, histology, and physiology of the teeth gave us a realization of the intimate relation existing between the teeth and the body. The advancement made in pathology led us to believe that it might be possible, and even probable, that dental lesions had certain harmful effects upon the constitution, besides those that would come from poorly masticated food. The study of bacteriology, which is still in its infancy, is now adding evidence to prove that diseased mouth conditions bring about systemic disorders.

This increase of knowledge has been, and necessarily must be, slow, while mechanical inventions and operative skill have been extremely rapid, particularly in our own country. The ability of American dentists in saving and keeping the teeth in the mouth is known throughout the world. In spite of the wonderful ability in repairing and saving the teeth, we are confronted by the fact that it is impossible for the dentists of the United States to repair and save the decayed teeth of all the people. What is to be done? The only answer that can be given is: we must study and teach preventive measures. We all know that if the teeth and the mouth are kept clean, the teeth are less likely to decay. We do not know as yet what factors there are that will produce decay in teeth that are clean; some claim that a clean tooth can not, and does not, decay; some claim that teeth may decay in the cleanest mouth. Be that as it may, none deny that cleanliness is not only desirable, but it is one of the greatest factors in the prevention of dental lesion. None will deny that the presence of decaying bone tissue in the mouth, being mixed and swallowed with the food, is a detriment to sound and healthy bodies. We also know that reflect nerve irritation can arise, and often does arise, in a decayed tooth affecting nerves and nerve centres, and bring about serious results. The far-reaching influence of reflex nerve action we are only commencing to understand.

For the dental profession to be of the greatest value to the community at large, it is important and absolutely necessary to gain the active co-operation of the people to bring about conditions that will aid in the prevention of dental lesions.

This can be done in three ways:

1. Through the press, magazine articles, and books written in simple and popular style.

2. Through the co-operation of Municipal, State, and Government officials.

3. Through illustrated lectures. Of these, the one having the greatest educational value is the lecture, particularly that which is illustrated. Books, magazine articles and newspapers are of great value, but they have not the personal quality which counts for so much in all educational work.

The speaker is able to pause a moment and explain more in detail here and there just as he receives the "feel" from his audience. He soon learns to be able to tell at once when his audience is not quite certain about some point, and he quickly responds and explains more fully. He can change readily from the argumentative form to the questioning form, to which the answers are perfectly obvious. In this way, and with the aid of clear and simple illustrations, strong and lasting impressions will be made upon the minds of all.

I have written to some of my friends asking their opinion on the value of oral hygiene lectures to the public, and will now read some of the answers:

The responsibilities of the dental profession rest upon the knowledge of the following facts:

1. That reparative work alone can not cope with the situation.

2. That much of the reparative work is destroyed and rendered useless by the same conditions which brought about the need of reparative work in the first place.

3. That dental lesions not only impair the proper preparation of food for digestion, but they bring about systemic disturbances which, in many cases, lead to serious illness or even death.

4. That people in general are ignorant of these truths.

5. That people in general do not realize it is within their power to prevent, in large measure, the establishment of these conditions in their own mouths.

6. That dental lesions can be greatly lessened, and in some cases entirely eliminated, by proper care and attention.

7. That preventive measures can be used by all, and that none are too young to learn how to practice them and understand the reasons why they should practice them.

8. That none are better qualified to teach how to do these things, and why to do them, than the members of the dental profession.

In an examination of over 1,000 cases, an average of five cavities to each patient was found. Following along this line of argument, there should be five hundred million cavities in the mouths of the one hundred million population in the United States.

Allowing one-half hour as being the time necessary to prepare and fill one cavity, this would mean it would take two hundred and fifty million hours to attend to this work. If we take out holidays and Sundays, allowing thirty days for vacation and illness, and counting eight hours a day's work, we will find it will take one hundred and twenty-five thousand dentists one year to do this work. There are only forty thousand dentists in the United States.

If one per cent. of this work needed to be done over again, owing to the neglect of the mouth or to other causes, we would be confronted by five hundred thousand cavities, and if any of these involved pulp removal and root filling, the allotment of one-half hour for each cavity would be far too small.

But even if all this tremendous amount of work was done, we still would find that the cause which brought into existence the need of this work had not been removed.

It is practically impossible for every dentist to give to his patients a talk upon the value of mouth hygiene and how to practice it. It requires at least one hour to be able to give, with the aid of illustrations, a comprehensive talk upon this subject so that a person may understand in a logical and intelligent manner the importance of sound teeth and clean mouths and the systemic changes which are brought about through ignorance or neglect. Merely to tell a patient how to brush his teeth does not answer the need of the present time. People should know, and they have a moral right to know, that many systemic disturbances are brought about by mouth conditions. They should know, and be made to understand, that our profession can do but little without their co-operation and intelligent understanding of the importance of the subject. They should know that the physical growth and the intellectual growth of the child are jeopardized through neglect of mouth conditions.

These are some of the answers I received in reply to my request: "What is the responsibility of the dental profession in relation to the oral hygiene campaign?"

I believe it can be justly claimed by those who started and have worked for the oral hygiene campaign that certain results have already been attained such as the following:

The establishing of Dental Clinics in many of the Public Schools of Europe and in our country.

The recognition by the International School Hygiene Association of the importance of dental attention for school children.

The establishment of an infirmary entirely devoted to the dental welfare of children. I refer, of course, to the Forsyth Institute of Boston.

That one of the largest life insurance companies in America, the Metropolitan Life Insurance Company, have established a dental division for their employes. Here only prophylactic work and examination is being done. The teeth needing reparative work of any kind are marked on a slip and the patient told to go to his dentist to have the work done. You may be interested in hearing the report for the first three months:

We have recently learned that Rochester is to have an infirmary devoted exclusively to the dental welfare of the children.

Realize, as we must, the importance of dental hygiene and what may be done in the way of public education through the press and public lectures, the responsibility of the dental profession is very great. All state and local societies should use every effort to encourage their members to give such talks. As officers of the societies have greater influence than individual members, they should be the ones to visit the state and city officials, particularly those of the Educational Department, and gain their cooperation in carrying on this work.

The New York Department of Education, through its lecture bureau, has for many years given lectures on oral hygiene, and last spring Dr. C. Ward Crampton, the director of physical training of the board, inaugurated a dental hygiene week. I have great pleasure in reading some of the notices sent out by Dr. Crampton to presidents of mothers' clubs, district superintendents, and principals of schools.

The first and second district dental societies of New York State presented banners to the school whose children gave the best tooth-brush drill. These drills were held in Central Park and in Prospect Park.

At Bridgeport, Connecticut, Dr. A. C. Fones and his co-workers have started prophylactic work in the Public Schools, and their first report is of great interest. Six thousand seven hundred and sixty-eight children received prophylactic treatment; some received one treatment, some two, and some three. The total number of phophylactic treatments given was fourteen thousand three hundred and forty. The supervisors gave tooth-brush drills to twelve thousand five hundred and forty-six children. Dr. Fones and Dr. Strang gave stereoptican lectures to seven thousand four hundred and forty-seven children.

This can be done, and should be done, in every city in the country, and it will be done when the members of our dental societies realize the importance of the work and the responsibilities resting upon them.—*Oral Health.*

